# Essays in Heart and Lung Disease

**Published:** 1895-12

**Authors:** 


### Reviews of Books.

---

*Essays in Heart and Lung Disease.* By ARTHUR FOXWELL, M.D. Pp. xiv., 476. London: CHARLES GRIFFIN AND COMPANY, LTD. 1895.

These essays have for the most part been previously published, but are here collected in book form and revised up to date. They are mostly on various points relating to pulmonary phthisis, and they give us a modern author's views on the prognosis and curability of the disease by various antiseptic and climatic modes of treatment. He concludes his account of hæmoptysis with the admonition: "Above all things let the thought of morphia be ever in your minds." He adduces much evidence in favour of iodoform in tubercular disease, and apparently prefers it to creasote and guaiacol, with which drugs his experience has been singularly unfortunate; for he states that "creasote acts as a gastric irritant in any quantity beyond a daily dose of ♏ 6." We cannot but think that a further experience of such drugs will greatly modify the views expressed on page 426.

The condition of the heart in debility and anæmia is a subject to which Dr. Foxwell has given much attention. His Ingleby lectures on this subject are reproduced (pp. 299 to 374), and are well worthy of careful study.

